# Quantitative beam optimization for radiotherapy of peripheral lung lesions: A pilot study in stereotactic body radiotherapy

**DOI:** 10.1002/acm2.70029

**Published:** 2025-02-22

**Authors:** Hamed Hooshangnejad, Jina Lee, Leslie Bell, Russell K. Hales, Khinh Ranh Voong, Sarah Han‐Oh, Kai Ding, Reza Farjam

**Affiliations:** ^1^ Department of Biomedical Engineering Johns Hopkins University Baltimore Maryland USA; ^2^ Carnegie Center for Surgical Innovation Johns Hopkins University Baltimore Maryland USA; ^3^ Department of Radiation Oncology and Molecular Sciences Johns Hopkins University Baltimore Maryland USA

**Keywords:** beam optimization, integral dose, SBRT, therapeutic gain

## Abstract

**Background:**

To quantify beam optimization for stereotactic body radiotherapy (SBRT) of peripheral lung lesions.

**Method:**

The new beam optimization approach was based on maximizing the therapeutic gain (TG) of the beam set by minimizing the average physical depth of the lesion with respect to the beam's eye view (BEV). The new approach was evaluated by replanning the 25 SBRT lesions retrospectively to assess if a better plan is achievable in all aspects. Difference in 25 Gy isodose line volume (IDLV_25_ _Gy_), IDLV_20_ _Gy_, IDLV_15_ _Gy_, IDLV_10_ _Gy_, and IDLV_5_ _Gy_ between the two plan cohorts were calculated as a measure of plan size and fitted in a linear regression model against the changes in the lesion depth with respect to the BEV to assess the relationship between the changes in the treatment depth and that of the plan size.

**Results:**

Beam optimization achieved a better plan in all cases by lowering the depth of treatment with an average of % 20.03 ± 12.30 (3.66%–45.78%). As the depth of treatment decreases, the size of the plan also decreases. We observed a reduction of % 4.64 ± 4.55 (0.02%–21.58%, *p* < 3.8 × 10^−5^), %5.16 ± 5.54 (0.03%‐24.68%, *p* < 0.005), %6.46 ± 6.95 (−1.35%‐29.05%, *p* < 0.009), %12.83 ± 9.06 (0.89%–37.65%, *p* < 0.0001), and %14.01 ± 9.87 (1.43%–41.84%, *p* < 4.5 × 10^−6^) in IDLV_25_ _Gy_, IDLV_20_ _Gy_, IDLV_15_ _Gy_, IDLV_10_ _Gy_, and IDLV_5_ _Gy_, respectively.

**Conclusion:**

Physical depth of the lesion with respect to the BEV is inversely proportional to the TG of a beam‐set and can be used as a robust and standard metric to select an appropriate beam‐set for SBRT of the peripheral lung lesions. Further evaluation warrants the utility of such concept in routine clinical use.

## 1 INTRODUCTION

Lung cancer is a leading cause of cancer death in the United States with estimated new cases of 253 000 and death of 130 000 in 2024.^1^ From all types of lung cancer, non‐small cell lung cancer (NSCLC) comprises the majority of cases.^2^ For localized NSCLC, the overall 5‐year relative survival rate is nearly 65%. For advanced disease, the 5‐year relative survival rate may drop to 9%.^3^ Among various treatment modalities, radiotherapy (RT) has been shown to be the only modality for which there is indication in all stages of lung cancer and can be part of the disease management in nearly 77% of the patients.^4^ Stereotactic body radiation therapy (SBRT) is the standard of care in patients with inoperable early‐stage disease including those with poor pulmonary function at baseline^5^ and has been suggested that may lead to a better overall survival (OS) compared to surgery in those with operable disease.^6^ RT may also be given concurrently or sequentially with chemotherapy in locally advanced disease^7^ or combined with targeted therapy such as immunotherapy.^8^ Patients with early‐stage disease may also benefit from these treatment combinations as such patients still have high rates of relapse after definitive resection or SBRT, thus, there may be a need for post‐resection or post‐SBRT consolidation therapy.^9^

In advanced disease, RT is mainly limited by radiation‐induced lung injuries (RILI) which includes radiation‐induced pneumonitis (RP) and the subsequent radiation‐induced fibrosis (RF) which can affect 15–40% of patients.^10^ In early‐stage disease where lesions are small and usually treated with ablative dose, the radiation‐induced toxicity is minimal but cumulative dose from multiple treatments can increase the chance of lung injuries or limit the future treatments if needed. The total lung dose is the main factor in causing RP. Various radiation dosimetric parameters including the mean lung dose (MLD), V60 Gy (volume of lung receiving 60 Gy), V20 Gy, and low dose volume such as V5 Gy have also been found to be correlated in development of radiation‐induced toxicity.^11^, ^12^, ^13^, ^14^, ^15^ Immunotherapy can also induce subsequent damage to normal tissue, a phenomenon called immune‐related adverse events (irAEs).^16^ It is believed that the rate of immune related lung injuries can be increased in combination with RT as each treatment modality can manifest some level of toxicity and the synergistic effect of the two could increase the chance of lung damage.^17^ Several pharmacological agents are currently under investigation to prevent and/or treat RP and RF such as protectors, modifiers, and mitigators of radiation‐induced lung toxicity. Angiotensin‐converting enzyme (ACE) inhibitors and angiotensin‐II receptor subtype 1 (AT‐1) antagonists have been shown to mitigate the radiation‐induced damage by targeting inflammatory and fibrogenic pathways in preclinical studies.^18^, ^19^, ^20^ Amifostine is traditionally used to attenuate renal toxicity and/or xerostomia during anti‐cancer chemoradiation therapy. Several clinical trials have shown its effectiveness to lower the rate of clinically apparent pneumonitis upon chemoradiotherapy of lung cancer patients.^21^, ^22^ Prophylactic use of inhalative corticosteroids has also been suggested to prevent radiation‐induced lung toxicity. However, despite encouraging preclinical results, clinical trials did not show efficacy of such agents in the prevention of RP and RF.^23^ In addition, studies have shown that increase in the time to treatment can adversely affect the treatment outcome in both early stage and advanced disease^24^, ^25^ requiring an administration of high‐quality treatment with an appropriate timing. Therefore, the best strategy to address the above issues could be investigating novel delivery techniques or standardized treatment planning approaches that can provide a robust and high‐quality treatment in a timely fashion.^11^

In recent years, improvement in treatment delivery techniques such as intensity‐modulated radiotherapy (IMRT) or volumetric modulated arc therapy (VMAT) played a significant role to enhance the therapeutic index of RT and reducing the RILI in lung cancer patients by improving the dose conformality^26^ and reducing the high dose volume in lung tissue. In contrast, these approaches increase the low dose volume which may be associated with different levels of toxicity. This issue is mainly due to the increase in the number of beam entry angles into the body. Therefore, we theorize that optimization of beam entry angles prior to plan optimization can help reduce the low‐dose volume in such treatment. In fact, due to the presence of large tissue heterogeneity in the lung region and variation in the target location, various beam angles have different impact on the therapeutic index of a treatment plan. This variation is more pronounced for non‐central lesions as the physical and water‐equivalent distance of the lesion to the beam entry varies significantly for various beam angles. When the target is small, the isocenter or the lesion center can be a good estimate of the lesion distance from the beam entry and the beam optimization can be achieved by focusing on the distance of the isocenter to the beam entry. However, this assumption is not valid for large lesion, hence more sophisticated metrics may be needed for such purpose. Since small lesions are usually treated with ablative dose, in this work, we primarily focused our attention on the optimization of the beam angles for the SBRT of the peripherally located lesions. So far, several literatures have shown the importance of optimal beam selection in reducing the organs at risk (OARs) dose in SBRT of the lung lesions.^27^, ^28^, ^29^ However, most of these works provide qualitative assessment such as use of couch kicks or non‐coplanar beams to reduce OARs dose. Therefore, the final beam arrangement could still depend on the planner's experience, vary person‐to‐person, and also need several attempts to find the best setting which can prolong the planning process as well. In this work, we aim to develop a quantitative metric to achieve a robust and standard beam arrangement to develop a high‐quality treatment plan for ablative RT of peripheral lung nodules.

## 2 MATERIALS AND METHOD

### 2.1 Quantification of optimal beam selection

In conventional SBRT with VMAT, depending on the location of the lesion, a set of partial or full arcs is designed by the planner in an optimization setting and then several clinical objectives are defined and updated interactively based on the clinical goals to come up with a desired treatment plan. It has been shown that optimal beam selection prior to plan optimization can help reduce the OARs dose and achieve a better plan.^27^, ^28^, ^29^ To quantify this process, we follow the below procedure.

For an isocentric treatment, the dose deposition and fall‐off are formulated by the tissue maximum ratio (TMR).^30^ A lesion gets the highest relative dose compared to its surrounding OARs when it is located at the depth of maximum TMR (d_max_). Hence, the closer a lesion to the d_max_, the higher the therapeutic gain (TG) of a beam where the TG is defined as follows:

(1) $$TG\left(b_{\theta }\right)=\frac{\int TMR_{tumor}\left(b_{\theta }\right)dl}{\int TMR_{OARs}\left(b_{\theta }\right)dl}\;$$

In the above, *b*_θ_ denotes the beam at the angle θ and OARs represent any voxel beyond the tumor boundary on the beam trajectory line. Similarly, we can define the TG for an arc as follows:

(2) $$TG\;\left(Arc\left(\theta_{start},\theta_{stop}\right)\right)=\int_{\theta_{start}}^{\theta_{stop}}TG\left(b_{\theta }\right)\;d\theta$$

In treating the peripheral lung lesions, partial arcs are usually preferred to avoid entering the contralateral lung. Also, to achieve acceptable conformality around the target, the length of the arcs is often set to be equal or greater than a half arc. Hence, we aim to find a set of half arcs with maximum TG for a lesion.

Since the chest wall thickness is often more than 2 cm, it can provide adequate buildup to establish electron equilibrium for a photon beam with an energy of 6MV. This also results in treating the lesion with a TMR < 1. Therefore, the closer a lesion to the chest wall, the higher the TG of the beam. In other word, the TG of a beam is inversely proportional to the physical distance of the lesion to the beam entrance. For small lesions where the distance of lesion center or isocenter to the beam entrance can be a good estimate of lesion distance to the beam entrance, we may write

(3) $$TG\left(b_{\theta }\right)\propto \frac{1}{d_{ph,\theta }\left(iso\right)}$$

**FIGURE 1:**
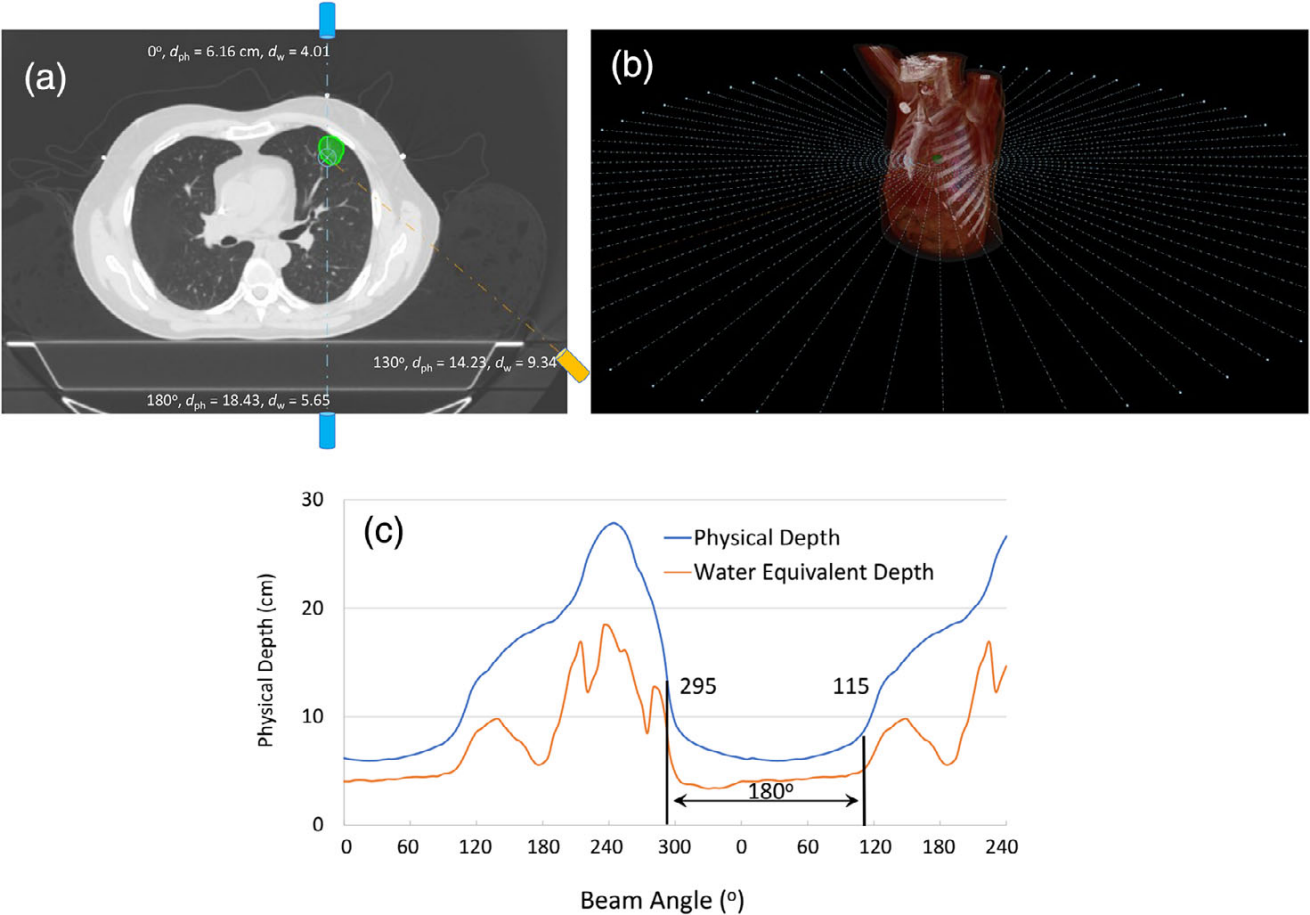
TG for a beam angle is inversely proportional to the physical depth of a lesion to the bean entry at the patient surface. (a) Various beam angles with their corresponding physical and water equivalent depths; b0 and b180 have the highest and lowest TG among these three beams. Although b130 has higher water equivalent depth compared to b180, it has higher TG than b180 as it treats less lung tissue before the beam reaches the target. (b) and (c) To determine the best arc arrangement, the plot of physical depth versus beam angle for the lesion is estimated by placing 72 evenly‐spaced beams around the lesion. The distance of the lesion center to the patient surface indicates the physical depth at that angle. The optimal arc set to treat the lesion is the 180 arc‐set with the lowest area under the curve which will be a set of 295–115 partial arcs for this case. TG, therapeutic gain.

Where in the above, $d_{ph,\theta }\left(iso\right)$, denotes the physical distance of the isocenter at an angle θ to the patient surface. Please note that due to the presence of lung tissue, water equivalent depth of a beam at certain angle could be lower than that of the other angles but this does not guarantee that the TG at that angle is also higher since the lung tissue is initially treated before that beam reaches the target and this lower the TG of that beam, Figure 1a. Finally, the TG of a beam set is inversely proportional to the average physical depth of the isocenter to the beam entry as follows, Figure 1b:

(4) $$TG\left(Arc\left(\theta_{start},\theta_{stop}\right)\right)\propto \frac{\theta_{Stop}-\theta_{Start}}{\int_{\theta_{start}}^{\theta_{stop}}d_{ph,\theta }\left(iso\right)}$$

Figure 1a shows a parallel opposed beams and one independent beam angle with the corresponding values of physical and water equivalent depths. As shown, *b*_0_ has the highest TG and b_180_ has the lowest TG for the lesion shown in this Figure. Also, although b_130_ has higher water equivalent depth than b_180_, it has higher TG due to its lower physical depth. Figure 1c, shows the plot of physical depth versus beam angles for this lesion. Figure 1c is estimated by placing certain number of (72 in this work) evenly‐spaced beams, Figure 1b, around the lesion and computing the distance of the lesion center to the patient surface at each direction. The optimal arc set to treat the lesion is the set of half arc with the lowest area under this curve which will be a set of 295–115 partial arcs for this case.

### 2.2 Treatment planning

Twenty‐five patients who had peripherally located lung lesions and were recently treated with SBRT were included in this IRB‐approved study, Table 1. Planning target volume (PTV) ranged between 5.11–71.52 cc (24.20 cc ± 17.11). Lesions were treated with a prescription dose of R_x_ = 10 Gy * 5 (*N* = 20), 12 Gy * 4 (*N* = 4) and 12.5 Gy * 4 (*N* = 1). For each target, a plot of physical depth versus beam angle was generated using an in‐house script to provide the 180 arc‐set with minimum area under the curve as the optimal beam set. Table 1 also shows the average physical depth of the lesion with respect to the original and optimal beam sets for each lesion. Each case was re‐planned using the new beam‐set with the aim that a superior plan is achieved compared to the clinical plan used to treat the patient and evaluated in our very rigorous peer review process prior to the study. A superior plan is a plan with better coverage, conformity index and OARs sparing for all normal tissue compared to the clinically approved plan. We designed the study that way to assure that improvement in one aspect is not the result of compromise on other aspects of the plan. Also, we accepted a plan only if the total number of monitor unit (MU) for the new plan was lower than the initial plan. Since each round of optimization can increase the total MUs and plan improvement could happen with the cost of overmodulation, we limited the new plan with the original MUs to assure that any possible improvement is the result of optimal beam selection rather than overmodulation. Table 2 shows the clinical objectives used for this study. Also, in our institution, we used RTOG conformity indices^31^ to assess the plan conformality as follow:

$$CI_{RTOG}=\frac{V_{RI}}{TV},\;\mathrm{RTOG}\;\mathrm{Conformity}\;\mathrm{Index}$$

(5) $$Q_{RTOG}=\frac{I_{min}}{RI},\;\mathrm{RTOG}\;\mathrm{Quality}\;\mathrm{of}\;\mathrm{Coverage}\;\mathrm{Index}$$

$$GI=\frac{V_{50\%rx}}{V_{rx}},\;\mathrm{Gradient}\;\mathrm{Index}$$

$$\;H_{RTOG}=\frac{I_{max}}{RI},\;\mathrm{RTOG}\;\mathrm{Homogeneity}\;\mathrm{Index}$$

Where in the above, TV denotes the target volume, V_RI_ is the prescription isodose volume, I_min_ and I_max_ is the minimum and maximum dose in the target, respectively, RI indicates the prescription dose, V50%rx is the volume of 50% prescription dose and V_rx_ shows the volume of the prescription dose. Planning was done using RayStation^32^ (RaySearch Laboratories Inc, Clinical Version 2023B) treatment planning system. The clinically approved plans were done using a set of two partial arcs (clockwise [cw] and counter clockwise [ccw]) with 2° gantry spacing. 6 MV flattening free filter (fff) energy was used in all cases. In the new plan, each arc is replaced with the new arc but with the initial setting. Dosimetrists who did the clinical plans were blinded about this study.

**TABLE 1:** Characteristic of the patient and lesions used in this study.

							${\bar{d}}_{ph}$(cm)	
No.	F/M	Location	Primary diagnosis	TNM staging	Dose (Rx)	PTV (cc)	Initial beam‐set	Optimal beam‐set
1	F	RLL	SCCa of epiglottis	Metastatic lung	1000 * 5	16.01	12.56	7.38
2	F	RLL	Lung Adenocarcinoma	T1NXM0	1000 * 5	28.23	8.4	5.91
3	F	RUL	NSCLC	T1bN0M0	1000 * 5	30.45	10.88	7.66
4	M	RLL	Thymic carcinoma	Metastatic lung	1000 * 5	25.06	15.26	13.25
5	F	RLL	SCLC adenocarcinoma	T1N2M0	1000 * 5	9.64	17.44	12.22
6	F	LLL	SPNs	Solitary Nodule	1000 * 5	11.5	9.28	8.13
7	F	LUL	Lung adenocarcinoma	T1NXM0	1000 * 5	49.39	9.13	8.13
8	M	LUL	SPNs	Solitary Nodule	1000 * 5	25.31	11.46	10.3
9	F	LUL	Lung adenocarcinoma	T1NXM0	1000 * 5	29.31	12.28	10.75
10	F	LUL	Lung adenocarcinoma	T1N0M0	1200 * 4	16.84	8.24	6.88
11	M	RUL	Colorectal cancer	Metastatic lung	1000 * 5	10.26	10.66	9.47
12	M	RUL	SCLC	T1N0M0	1200 * 4	17.04	9.9	8.81
13	M	LUL	Sarcoma	Metastatic lung	1000 * 5	8.71	9.37	6.39
14	F	L apical	Lung adenocarcinoma	T4N2M1a	1000 * 5	10.02	11.2	10.79
15	F	LLL	Lung adenocarcinoma	T2aN2M1b	1000 * 5	7.68	6.41	6.083
16	F	RML	SPNs	Solitary Nodule	1000 * 5	14.74	10.12	9
17	M	LUL	Lung adenocarcinoma	T2bN0M0	1000 * 5	53.77	11.09	8.666
18	M	RUL	NSCLC	T2N0M0	1000 * 5	51.46	9.48	8.41
19	M	LUL	Lung adenocarcinoma	T1N0M0	1250 * 4	19.83	9.91	7.05
20	F	RLL	Lung adenocarcinoma	T1N0M0	1200 * 4	12.06	6.93	6.38
21	M	RLL	NSCLC	T1CN0M0	1000 * 5	71.52	15.39	11.66
22	F	RLL	N/A	Solitary nodule	1000 * 5	5.11	7.26	4
23	M	RLL	Urothelial carcinoma	Metastatic lung	1000 * 5	16.3	8.3	4.5
24	M	LUL	GE Adenocarcinoma	Metastatic lung	1200 * 4	22.71	13.1	10.83
25	M	LLL	SPNs	Solitary nodule	1000 * 5	42.06	7.29	6.69

*Note*: The average physical depth of the lesion for the initial and optimal beam arrangement are also shown for each lesion.

Abbreviations: ${\bar{d}}_{ph}$, Average physical depth; F, Female; GE, gastrointestinal; LLL, left lower lobe; LUL, left upper lobe; M, Male; NSCLC, non‐small cell lung cancer; PTV, planning target volume; RLL, right lower lobe; RUL, right upper lobe; Rx, prescription; SCCa, Squamous cell carcinoma; SCLC, small cell lung cancer; SLNs, solitary pulmonary nodules; TNM, tumor node metastasis.

**TABLE 2:** Dosimetric indices used to define the clinical goals in this study.

Structure	Dosimetric index (Unit)
PTV	V100 (%)
	RTOG quality of coverage
	RTOG homogeneity index
	Mean (cGy)
GTV	V100 (%)
Conformity indices	RTOG conformity index
	Gradient index
	Ratio of Dmax at 2 cm to Rx dose (%)
Lung	MLD, cGy
	V20 Gy (cc)
	V10 Gy (cc)
	V5 Gy (cc)
Chest wall	V30 Gy (cc)
	V45 Gy (cc)
Spinal cord	Max dose (cGy)
Skin	Max dose (cGy)
Pericardium	Max dose (cGy)
Esophagus	Max dose (cGy)
Airways	Max dose (cGy)

Abbreviation: GTV: gross tumor volume; MLD; mean lung dose; PTV: planning target volume.

## 3 EVALUATION

To evaluate the performance of the beam optimization approach, we initially assessed each new plan to verify that if all clinical objectives achieved a comparable or better value compared to the initial plan. Then, we calculated the isodose line volumesIDLVs of 25 Gy (IDLV_25_ _Gy_), 20 Gy, 15 Gy, 10 and 5 Gy for both initial and new plans as a measure of plan size for each isodose line level. The volumetric change of each isodose line level was then calculated between the initial (i) and new (n) plans as follows:

(6) $$\begin{array}{ccc} & & \%\Delta \;IDLV_{x}=\frac{\left(IDLV_{x,i}-IDLV_{x,n}\right)}{IDLV_{x,i}}\;*100,\\ & & x=25,\;20,\;15,\;10\;and\;5 \end{array}$$

For each patient, the difference between the average physical depth of the lesion with respect to the treating beam set was also calculated between the initial (i) and new plan (i) as follows:

(7) $$\%\Delta {\bar{d}}_{ph}=\frac{\left({{\bar{d}}_{ph}}_{i}-{{\bar{d}}_{ph}}_{n}\right)}{{{\bar{d}}_{ph}}_{i}}*100$$

For each isodose line level, the scatter plot of $\%\Delta IDLV_{x}$ versus $\%\Delta {\bar{d}}_{ph}$ was drawn and fitted to a linear regression model to assess the relationship between changes in the depth of lesion from the beam's eye view (BEV) and changes in the plan size at that particular dose level. Similar analysis was also performed for the lung tissue in a way that changes of MLD between the initial and new plans (%ΔMLD), V20 Gy (%ΔV20), V10 Gy (%ΔV10), and V5 Gy (%ΔV5) were also plotted against the $\%\Delta {\bar{d}}_{ph}$ and fitted to a linear regression model to assess the relationship between the changes in the depth of lesion with respect to the BEV and changes in the lung dosimetric indices. Dosimetric statistics of the above metrics were also compared between the two plan cohorts. Statistical student paired *t*‐test was performed when comparing the two plan cohorts.

## 4 RESULTS

**FIGURE 2:**
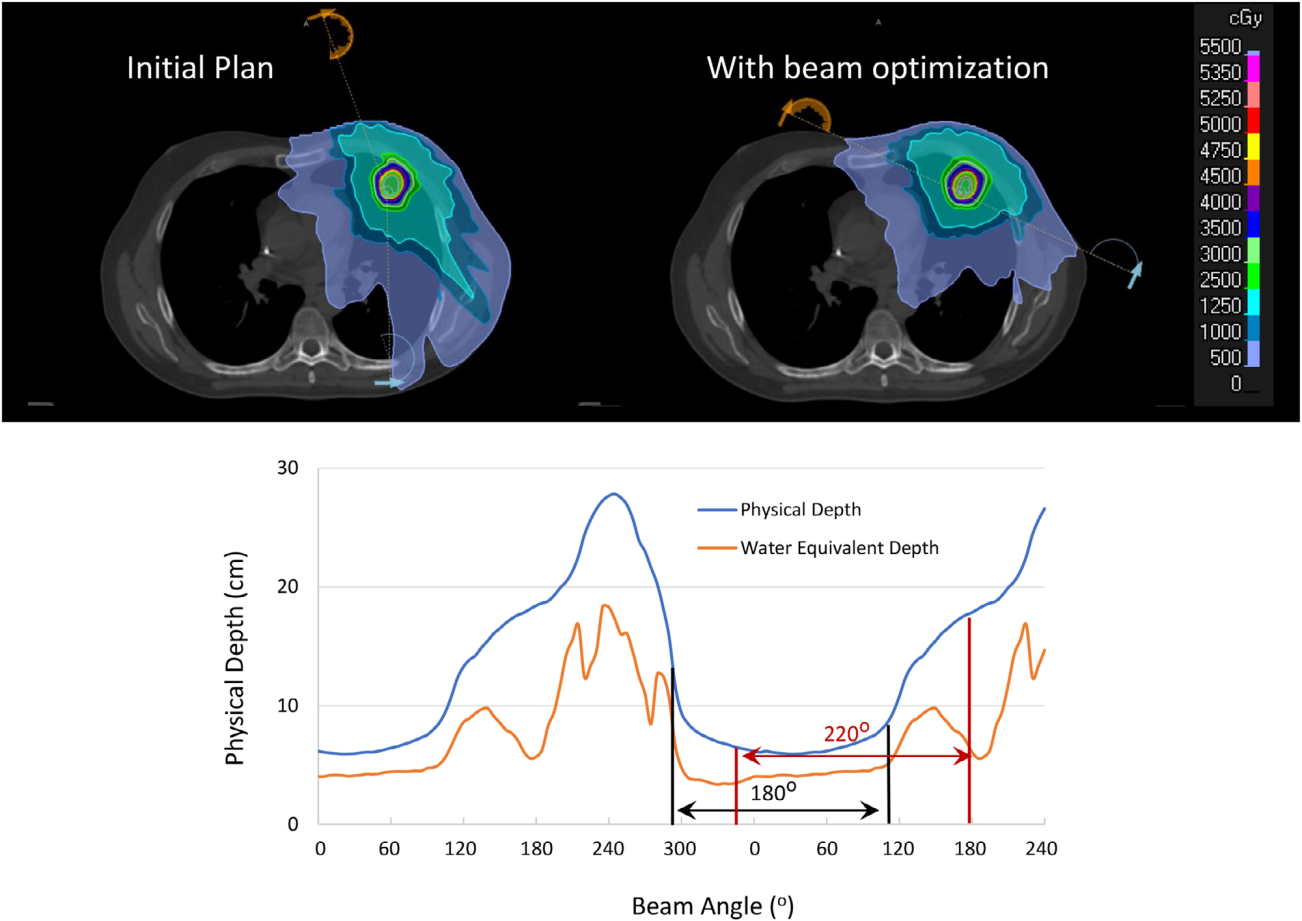
The dose distribution between the initial and plan with beam optimization. The physical depth of the lesion with respect to beam's eye view was decreased from 9.37 to 6.39 in the new plan. In the initial plan, IDLV25Gy = 52.21 cc, IDLV20Gy = 87.34 cc, IDLV15Gy = 166.55 cc, IDLV10Gy = 395.53 cc, and IDLV5Gy = 943.09 cc; In the new plan IDLV25Gy = 48.6 cc, IDLV20Gy = 78.33 cc, IDLV15Gy = 138.13 cc, IDLV10Gy = 268.89 cc, IDLV5Gy = 677.83 cc. The initial plan was done with a beam set of 340–179 (cw, ccw) and the new plan was done with a beam set of 295–115 (cw, ccw). cw: clockwise; ccw: counter clockwise;IDLV, isodose line volume.

**FIGURE 3:**
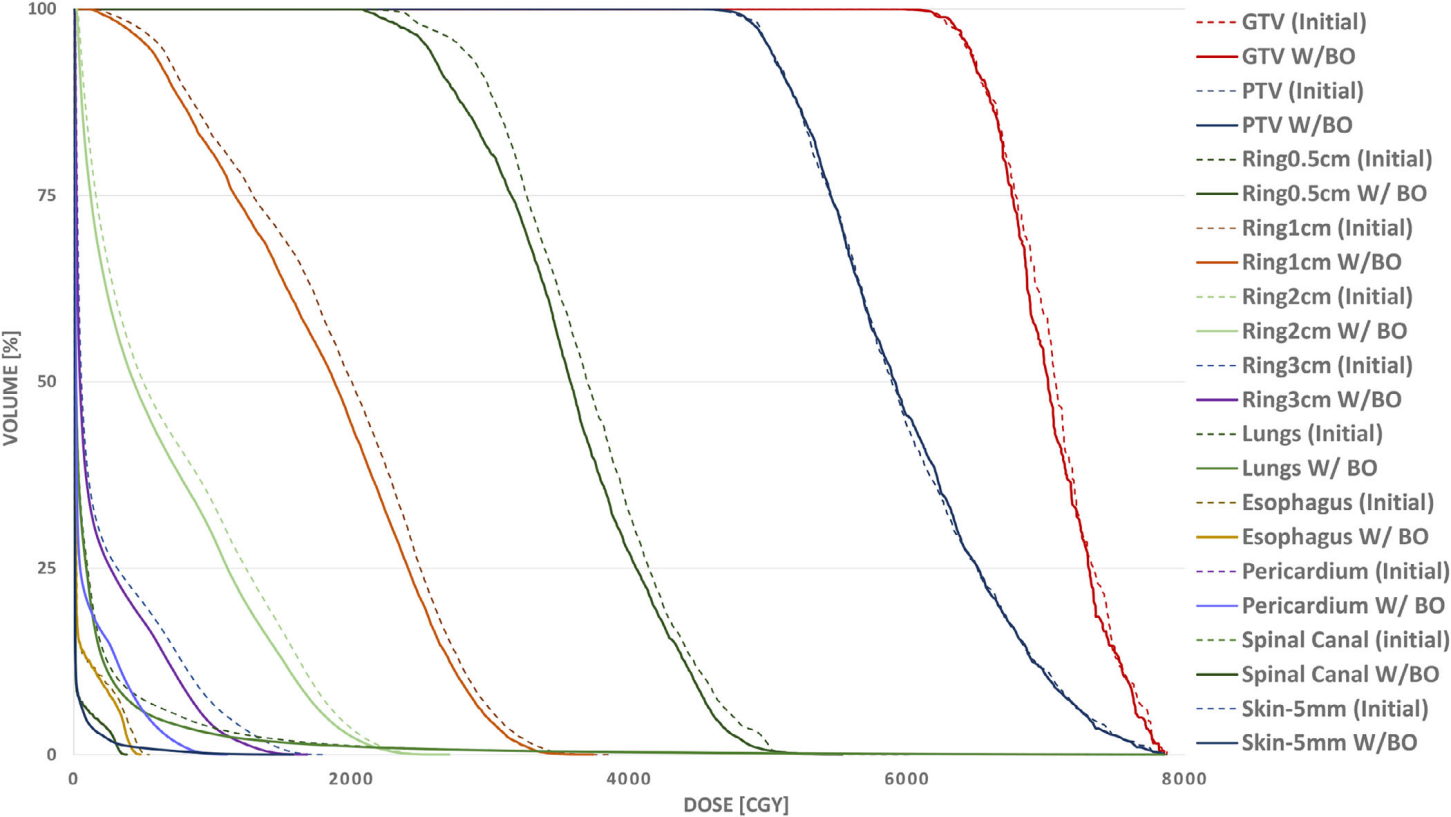
The DVH between the two plans shown in Figure 2. To better illustrate different rates of dose reduction as a function of distance from the target in the two plans, the DVH of inter‐connected rings with diameters of 5 mm, 1 , 2, and 3 cm are also shown. Ring0.5 mm encompasses the target while Ring1 cm encompasses Ring0.5 cm and so on. The numeric value of different dosimetric indices used for plan comparison of the two plans are also shown in Table 3. DVH, dose volume histograms.

Beam optimization achieved a better plan for all patients in our study. In other words, with the same or better coverage, a plan with equal or better OARs sparing and lower MU was achieved for all lesions using the beam optimization approach. Figures 2 and 3 illustrate the initial and plan with beam optimization along with the dose volume histogram (DVH) comparison between the two plans for the lesion shown in Figure 1 along with the position of the arc set for each beam arrangement on the physical depth versus beam angle curve. Using beam optimization, the average physical depth of the lesion with respect to the beam's eye view decreased by ∼31% (from 9.37 cm to 6.39 cm). Table 3 also shows a dosimetric comparison between the initial and new plan for this case. Please note that some of the OARs dose such as Spinal Cord max dose was very low for both plans but attempt was made to make sure that all OARs dose in the new plan was lower than the initial plan to assure that any possible improvement in the plan is not the result of dosimetric compromise to any other structures. It's worthwhile noting that better plan was achieved for this case while the total MU also decreased by ∼22%. Also, please note that a shorter arc set with fewer number of control points was used in the new plan.

**TABLE 3:** Dosimetric comparison between the initial and plans with beam optimization.

		Example shown in Figure 2		Group comparison		
Structure	Dosimetric index (Unit)	Initial plan	W/ BO	Initial plan	W/ BO	*p* Value
PTV	V100 (%)	95.00	95.13	95.54 ± 1.10	95.64 + 1.12	0.021
	RTOG Quality of Coverage	0.923	0.927	0.897 ± 0.067	0.91 ± 0.035	0.13
	RTOG Homogeneity Index	1.572	1.569	1.59 ± 0.09	1.60 ± 0.09	0.47
	Mean (cGy)	6005	6006	6041.7 ± 134	6084.6 ± 136	0.001
GTV	V100 (%)	100	100	100	100	N/A
Conformity indices	RTOG Conformity Index	1.043	1.011	1.04 ± 0.04	1.03 ± 0.4	0.13
	Gradient Index	5.82	5.515	4.66 ± 0.73	4.47 ± 0.6	0.007
	Ratio of Dmax at 2 cm to Rx dose (%)	48.5	48.5	50.16 ± 6.9	47.8 ± 6.9	0.001
Lung	MLD, cGy	154	134	260 ± 103.8	244.1 ± 98	2.23 E‐09
	V20 Gy (cc)	60.07	54.63	113.8 ± 66	106 ± 60	0.0002
	V10 Gy (cc)	182.45	140.33	274.4 ± 129	233.5 ± 112	7.6 E‐09
	V5 Gy (cc)	373.3	291.95	477.6 ± 180	433.5 ± 173	3.4 E‐07
Chest wall	V30 Gy (cc)	5.71	4.6	10.5 ± 12	10 ± 11.8	0.0041
	V45 Gy (cc)	0.32	0.23	2.76 ± 3.9	2.6 ± 3.8	0.006
Spinal cord	Max dose (cGy)	375	361	759 ± 480	692 ± 435	6 E‐05
Skin	Max dose (cGy)	1538	1459	1554 ± 409	1506 ± 395	3.3 E‐05
Pericardium	Max dose (cGy)	1100	1074	715.1 ± 1028	666.1 ± 1024	0.003
Esophagus	Max dose (cGy)	810	760	802.8 ± 508	732.4 ± 460	0.0006
Airways	Max dose (cGy)	522	452	708 ± 698	635 ± 609	0.002
IDLV_25_ _Gy_ (cc)		52.21	48.6	109.2 ± 69	105.2 ± 67	3.8 E‐05
IDLV_20_ _Gy_ (cc)		87.34	78.33	174.4 ± 111	166.7 ± 108	0.0005
IDLV_15_ _Gy_ (cc)		166.55	138.13	321.9 ± 220	301 ± 202	0.0009
IDLV_10_ _Gy_ (cc)		395.53	268.89	699.4 ± 460	606.4 ± 376	0.0001
IDLV_5_ _Gy_ (cc)		943.09	677.83	1514.7 ± 771	1311 ± 664	4.5 E‐06
MU		3923	3032	3388.4 ± 760	3001.5 ± 582	0.0002

*Note*: The comparison includes a typical example shown in Figure 2 and the group comparison between all the 25 patients.

Abbreviations: cw: clockwise; ccw: counter clockwise; IDLV, isodose line volume; MLD, mean lung dose; MU, monitor unit.

**FIGURE 4:**
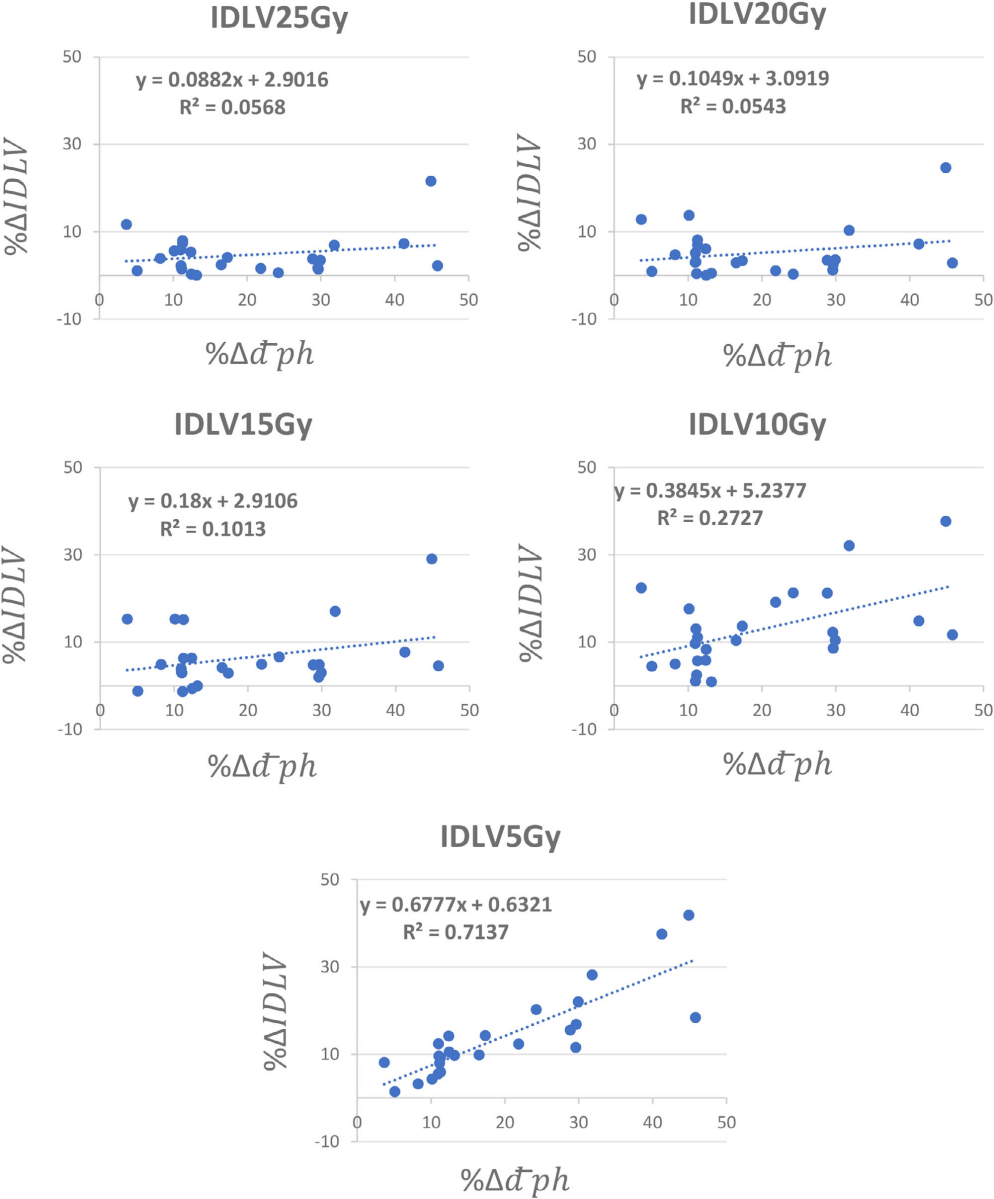
The scatter plot of $\%\Delta {\bar{d}}_{ph}$ versus $\%\Delta IDLV_{x},\;x=25,\;20,\;15,\;10,\;and\;5Gy$ between the initial and new plans for all 25 lesions in this study. As shown, as the difference between the physical depth of the lesion with respect to the beam's eye view increases, the difference between the volume of the lower isodose lines also increases. Hence, the beam optimization brings the lesion to a shallower depth leading to decrease the size of different isodose line levels. IDLV, isodose line volume.

**FIGURE 5:**
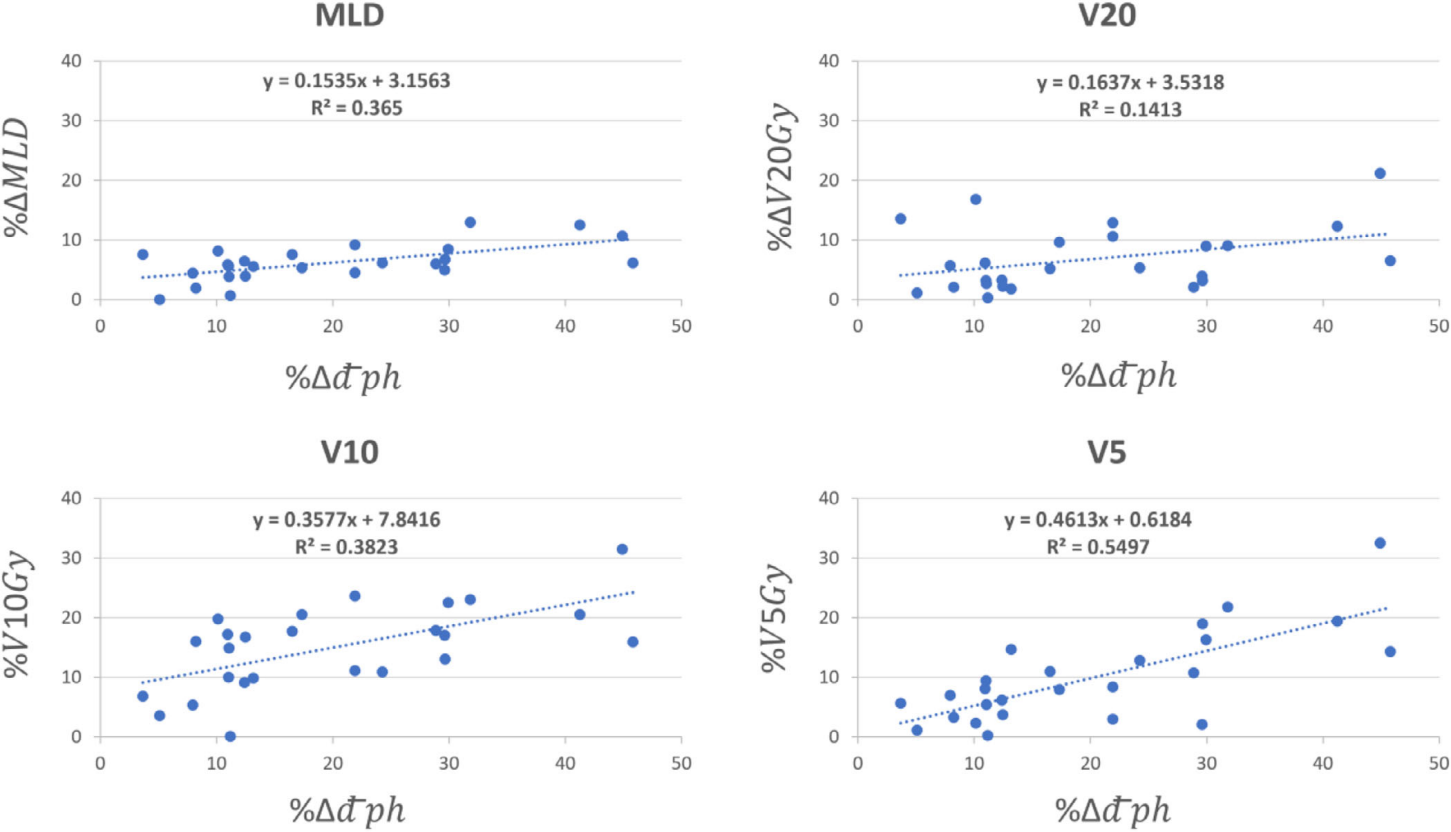
The scatter plot of $\%\Delta {\bar{d}}_{ph}$ versus %ΔMLD,%ΔV20Gy,%V10Gy,and%V5Gy between the initial and new plans for all 25 lesions in this study. It can be inferred from this figure that as depth of the lesion with respect to the beam's eye view increases, the difference in the MLD, V20 and V10 also increases. MLD, mean lung dose.

**FIGURE 6:**
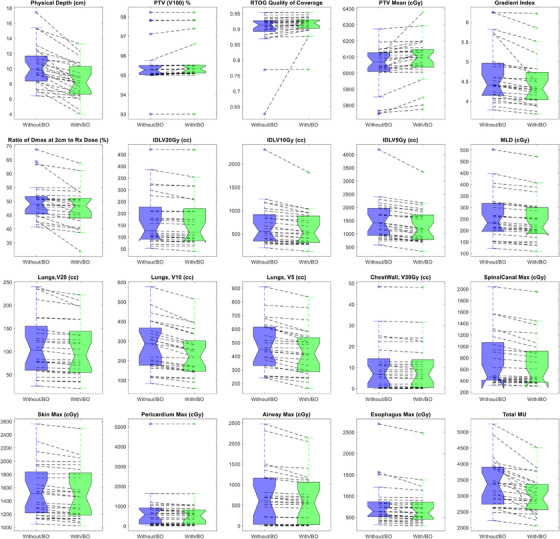
Boxplots of dosimetric indices between the initial and plans with beam optimization (new) for all 25 cases. Statistical comparison between the two plan cohorts is also shown in Table 3.

**FIGURE 7:**
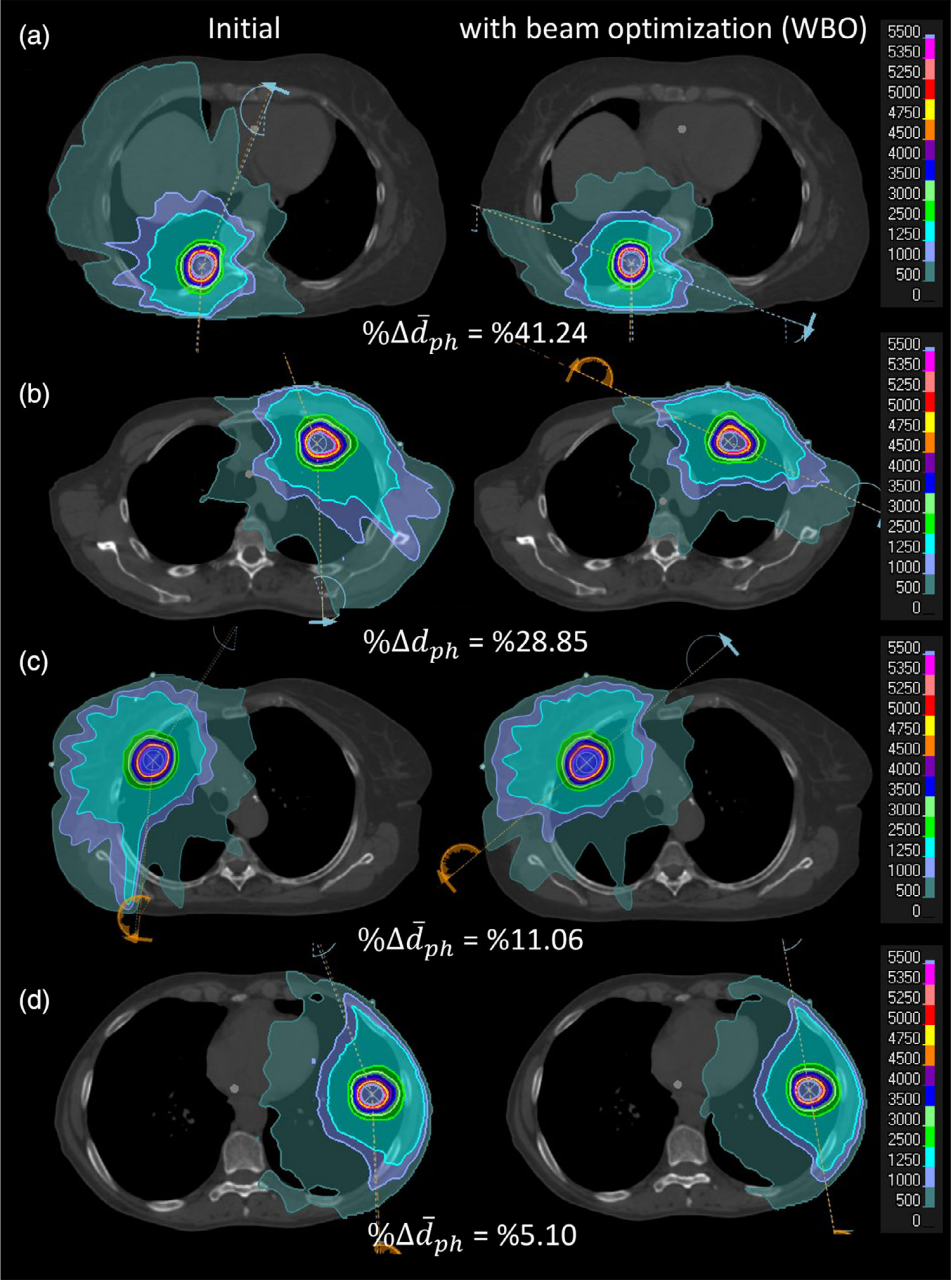
Illustration of initial and plan with beam optimization for cases with different changes in lesion depth with respect to BEV. As shown, as changes in the lesion depth between the two plans decreases, the difference between the size of the two plans also decrease. The IDLV5 Gy for each case is as follows: A‐Initial = 1415 cc, A‐WBO = 884 cc, B‐Initial = 1430 cc, B‐WBO = 1208 cc, C‐Initial = 1143 cc, C‐WBO = 1034 cc, D‐Initial = 736 cc, D‐WBO = 726 cc. BEV, beam's eye view; cw: clockwise; ccw: counter clockwise; IDLV, isodose line volume.

Figure 4 shows the scatter plot of $\%\Delta {\bar{d}}_{ph}$ versus $\%\Delta IDLV_{x},\;x=25,\;20,\;15,\;10,\;and\;5Gy$ between the initial and new plans for all twenty‐five lesions. The linear regression model parameters fitted to each plot were also shown for each isodose line level. As shown, as the difference between the physical depth of the lesion with respect to the beam's eye view increases, the difference between the volume of the lower isodose lines also increases. In other words, the beam optimization treats the lesion in a shallower depth leading to a decrease in the lower isodose lines’ volume. Figure 4 also shows that as we become more distant from the lesion, the impact of beam arrangement is more pronounced. On average, we observed a reduction of %4.64 ± 4.55 (0.02%‐21.58%, *p* < 3.8 × 10^−5^), %5.16 ± 5.54 (0.03%‐24.68%, *p* < 0.005), %6.46 ± 6.95 (−1.35%‐29.05%, *p* < 0.009), %12.82 ± 9.06 (0.89%‐37.65%, *p* < 0.0001), and %14.01 ± 9.87 (1.43%‐41.84%, *p* < 4.5 × 10^−6^) in the volume of IDLV_25_ _Gy_, IDLV_20_ _Gy_, IDLV_15_ _Gy_, IDLV_10_ _Gy_, and IDLV_5_ _Gy_, respectively. We also observed an average reduction of %20.03 ± 12.30 (3.66%–45.78%) in the ${\bar{d}}_{ph}$ between the initial and optimized beam sets. Similarly, Figure 5 shows the scatter plot of $\%\Delta {\bar{d}}_{ph}$ versus MLD, V20 Gy, V10 Gy and V5 Gy, and the regression model parameters between the initial and new plans, respectively. As shown, similar trends can be seen for various isodose line levels in the lung tissue as well. On average, we observed a reduction of %6.25 ± 2.95 (0%‐12.98%, *p* < 2.23 × 10^−9^), %6.98 ± 5.21 (1.14%–21.2%, *p* < 0.0002), %15.03 ± 6.94 (2.59%‐31.50%, *p* < 7.6 × 10^−9^), and %9.86 ± 7.54 (1.11%–32.49%, *p* < 3.4 × 10^−7^) in the MLD, V20 Gy, V10 Gy, and V5 Gy, respectively. Figure 6 shows the boxplots of dosimetric indices between the initial and plans with beam optimization (new) for all 25 cases. Statistical comparison between the two plan cohorts is also shown in Table 3. Finally, examples of new plans with different $\%\Delta {\bar{d}}_{ph}$ are shown in Figure 7.

## 5 DISCUSSION

In this work, we have proposed and evaluated a new approach to quantify the beam optimization prior to plan optimization in SBRT of the peripheral lung lesions. The new approach is based on maximizing the TG of the beam set by minimizing the average physical depth of the lesion with respect to the BEV. We assessed this approach primarily in lesions treated with SRBT because SBRT is usually applied to small lesions for which lesion center or isocenter is a good estimation of the lesion distance to the patient surface where beams enter. In this paper, we showed that lowering the depth of the treatment with respect to the BEV also lowers the overall integral dose of the plan.

To the best of our knowledge, this is the first attempt to develop a quantitative metric to determine the optimal beam arrangement for lung SBRT. Therefore, to overcome the lack of prior metrics for comparison, we evaluated this work using a set of plans that were used clinically and approved through a very rigorous peer review process in our center as the base cohort. Therefore, the base plan‐set had no bias regarding the value of our proposed metric. In our study, we assessed the effect of beam optimization by measuring the size of the plans at different IDLVs rather than comparing the dose in various OARs. This was mainly done because the OARs dose is a subjective metric for plan comparison and varies per user discretion and patient to patient. In other words, two plans might have similar size but substantially different dose distribution per planner's or physician's demand. Also, to make sure that beam optimization does not compromise the safety of any OAR in the new plan, we used the initial dosimetric objectives as the clinical goals and only accepted the new plan if these clinical goals were met. For example, it may be believed that new beam arrangements may increase the skin dose for some condition, but our results showed that beam optimization does not compromise the safety of any OARs in the new plan. In fact, the beam optimization decreases the size of the plan at different isodose line levels and it's the planner role to guide the optimizer how to distribute the dose. In the current dataset, we observed an average reduction of ∼%3.07 ± 2.69 (0.05%–8.94%, *p* < 3.3 × 10^−5^) in the skin maximum dose. Lastly, it's worthwhile to note that we accepted the new plans only if their total MUs were less than that of the initial plans. Since each round of optimization can increase the total number of MUs and could potentially improve the plan, we made this assumption to assure that any improvement in the plan quality was achieved due to beam optimization rather than overmodulation. We observed an average reduction of ∼%10.24 ± 10.24 (0.18%–46.06%, *p* < 0.0002) in the total MUs of the new plans. While reduction of MU may not sound as important as OARs dose reduction, this could significantly increase the machine throughput in slow‐delivery machine like MRI‐Linac^33^ and Cobalt‐device^34^ machines. The increased throughput has the potential to improve access to treatment in low‐ and middle‐income countries with limited resources. These countries are also projected to have a higher burden of cancer in the coming years. Efforts are underway to bring IMRT‐like treatment plans using cobalt devices at these resource‐limited settings.

One important feature of the beam optimization approach proposed in this work is that minimizing the average physical depth of the lesion with respect to the beam's eye view substantially decreases the lung tissue exposure before the beam reaches the target as well, Figures 2 and 7. Since RILI are the main limiting toxicity in RT of the lung lesions, this approach has the potential to lower the lung dose, Figure 5, and injuries systematically. Also, accurate dose calculation has been a historical challenge for lung SBRT due to the low density of lung structure and difficulty of small field dosimetry associated with the small targets. Studies have shown that different algorithms such as analytical anisotropic algorithm (AAA), collapse cone convolution (CCC) and Acuros XB (AXB) can show substantial dosimetric discrepancies in case of lung SBRT compared to Monte Carlo simulation and measurements.^35^, ^36^, ^37^, ^38^ One crucial point to consider in such cases is that the increased volume of lung in the beam trajectory will increase the dosimetric difference, an expected decrease in PTVD99% BED of 1.6% per 500 cc.^39^ Since in our proposed beam optimization, the beam geometry is defined in a way that lower lung tissue is exposed before the beam reaches the target, we hypothesize that this may also help reduce the uncertainty of dose calculation for PTV coverage. In addition, since many of the lung patients are treated with free breathing with possible effect of diaphragm or even pericardium movement in dose calculation, limiting the lung exposure could also mitigate the impact of internal motion in PTV dose calculation's uncertainty as beams hit the target before reaching the dynamic components of the thorax. Quantifying this feature is part of our future study.

One of the main challenges to obtain the ideal beam arrangement in our proposed approach is when the lesion seats posteriorly, Figure 7a, as we may need a set of full arcs with avoidance sector or split the fields into two half arcs to treat the lesion. The first scenario increases the chance of collision and for the second scenario, we may need to move the couch between the arcs, and this may add extra minutes to the delivery time or may require extra imaging scans. However, if the gantry can move freely around the patient, both ways can be followed seamlessly with an efficient delivery time. Therefore, developing a collision metric sounds essential for the posteriorly located lesions. In cases where the gantry can't rotate around the patient freely, beam optimization should include this as part of the process and make a balance between the collision and optimal beam selection. Luckily, the chance of collision increases when the lesion gets closer to the lateral part of the lung but in this case the role of posterior field in forming the optimal beam set decreases. In contrast, when the posterior lesion seats more medially, the chance of collision decreases and the impact of posterior field in forming the optimal beam set increases. One other solution for such a problem is to use an off‐axis isocenter for such lesions.^40^, ^41^ In new treatment modalities such as MRI‐Linac,^33^ PET‐Linac^42^ and Tomotherapy^43^ where off‐axis treatment is part of the routine practice, this approach can be easily applied. However, in regular Linac where off‐axis treatment is not routinely practiced, the potential drawbacks of such treatments should be evaluated comprehensively. While the plan quality of off‐axis treatment might be comparable to on‐axis treatments,^40^, ^41^ the accuracy of the dose calculation of the treatment planning system should be entirely assessed in RT of the thoracic malignancy where substantial tissue heterogeneity exists. Developing the collision metrics and assessing the off‐axis treatment for such lesions are currently underway in our institution. It's worthwhile to note that in the current study, we observed a significantly bigger change (*p* < 0.044) in the treatment depth of the posterior lesions (%24.68 ± 13.24) in contrast to anterior lesions (%15.00 ± 2.07) after beam optimization which can justify the effort of developing collision metrics and off‐axis treatment.

One other aspect of our proposed beam optimization approach is to use a set of half arcs for SBRT of the peripheral lung lesions. Such selection is important as a hemi arc includes beam angles in all directions which is crucial to achieve appropriate conformality for SBRT treatment. In one hand, any arc greater than 180° can increase the depth of treatment, due to inclusion of beam angles with larger physical depth into the beam set, and the plan size subsequently with no actual benefit. In other words, increasing the number of control points to a set of hemi‐arcs may not necessarily result in a better plan but could increase the total MU, the delivery time and more importantly the plan size. In the other hand, using arc shorter than 180 can compromise the conformality of the plan with an increase in the high dose volumes around the target. In addition, several studies have shown the benefit of couch kicks in the case of SBRT treatment.^29^ Since applying the couch rotation can also increase the chance of collision, we can perform a risk/benefit analysis by measuring the change in the lesion depth with respect to the BEV and make an appropriate decision in such cases. It's worthwhile to note that all of these can be achieved by eliminating the need to test different beam arrangement settings in the actual planning process which has the potential to reduce the planning time significantly. In addition, as automation now plays a key role in RT practice and especially in busy clinics, having a quantified metric in selecting the appropriate beam arrangement is a major step in developing a robust automated workflow. In this study, we assessed the beam optimization approach using a small sample size. Reassessing this concept with a larger plan cohort warrants the appropriateness of this technique for development of automated workflow and routine clinical use.

Beam optimization was done in this work based on the assumption that lesion center is a good estimate of the lesion distance to the patient surface where beams enter. This assumption can be true for small lesions but as the lesion size increases, more sophisticated metric will be needed to measure the distance of the lesion to the site of beam entry. In contrast, beam optimization might have bigger impact on larger lesions since the RT plans of such lesions are much bigger and might have shallower dose fall‐off. Therefore, a small reduction of plan size could generate bigger reduction in absolute volume of the plan compared to SBRT cases. It is also worthwhile to note that collision is of less concern for larger lesions due to flexibility of isocenter placement and higher tolerance of error on setup margin. Beam optimization of large lesions and non‐SBRT cases is underway in our center. Also, in this work, we assumed that all voxels beyond the PTV are equally important from the radiation‐induced toxicity perspective and lowering the overall integral dose and lung tissue exposure was the main goal of beam optimization. However, different OARs might be at different risk requiring unequally‐weighting optimization which can also be more pronounced in RT of large lesions where toxicity of other organs such as heart, esophagus, and so forth. is a limiting factor. In our future work, we focus on this concept as well. One other important note in that regard is that while VMAT is the treatment modality for SBRT cases,^44^ IMRT could outperform VMAT for larger lesions.^45^ Therefore, a smart decision on the selection of VMAT versus IMRT is also essential to design the best treatment for lung lesions. In this work, we have shown that depth‐angle curve is a good approximation of the TG‐angle curve for small lesions. Since the patient surface is approximately elliptical, the depth‐angle curve usually reaches a global minimum as shown in Figures 1 and 2 and as we become distant from this angle, the depth increases. Therefore, for small lesions with a unique global extremum, choosing a shortest half arc around that point will provide the best beam‐set. Hence, using IMRT with certain orientations for small lesions is just a subset of optimal beam angles used in VMAT with reduced degree of freedom in our beam‐set leading to an inferior plan compared to VMAT.^44^ In contrast, as the lesion size increases, the depth‐angle curve cannot represent the TG‐angle curve anymore as the lesion boundaries could be close to the patient surface at different orientations. In addition, the denominator of Equation (1) may also need to include different weighting for various OARs as well for larger lesions. Hence, the TG‐angle curve will likely have multiple extrema for large lesions for which IMRT could be a better choice compared to VMAT. In such cases, using split arcs around various local extrema could also be used for VMAT modality. In close, the introduction of TG concept not only provides the best beam arrangement for lung RT but also has the potential to guide us to select an appropriate choice of treatment modality leading to a more personalized RT for lung cancer patients.

## 6 CONCLUSION

In lung RT, the treatment outcome is mainly limited by the RILI which is highly correlated with its radiation exposure. This could also depend on the planner's skill and experience and varies significantly among different dosimetrists and even treating physicians. Treatment standardization has the potential to remove inter‐variability among various RT teams by generating a robust treatment plan in a timely fashion. We proposed and evaluated a new metric, treatment depth with respect to BEV, for optimal beam selection as the first step in standardization of lung SBRT to minimize the radiation‐induced lung toxicity and potentially decrease the time to treatment. The new metric can also be used to predict the quality of the plan in time. Further evaluation warrants the utility of such metric for routine clinical use and automated workflow.

## AUTHOR CONTRIBUTIONS

**Hamed Hooshangnejad**: Conceptualization; methodology; software; validation; formal analysis; writing—review and editing; visualization. **Jina Lee**: Conceptualization; methodology; software; writing—review and editing; visualization. **Leslie Bell**: Conceptualization; methodology; writing—review and editing; visualization. **Russel K. Hales**: Conceptualization; resource; writing—review and editing. **Khinh Ranh Voong**: Conceptualization; resource; writing; review and editing. **Sarah Han‐Oh**: Conceptualization; writing—review and editing. **Kai Ding**: Conceptualization; methodology; validation; writing—review and editing. **Reza Farjam**: Conceptualization; methodology; software; validation; formal analysis; investigation; writing—review and editing; visualization; supervision.

## CONFLICT OF INTEREST STATEMENT

The authors declare no conflicts of interest.
